# Supplementary material for: Metabolomic signatures associated with depression and predictors of antidepressant response in humans: A CAN-BIND-1 report
Source: Commun Biol. 2021 Jul 22;4:903. doi: 10.1038/s42003-021-02421-6 (PMC8298446; doi:10.1038/s42003-021-02421-6)
Supplement: Supplementary file 1 — Supplementary Information [file 42003_2021_2421_MOESM1_ESM.pdf]

Metabolomic signatures associated with depression and predictors of antidepressant response: A CAN-BIND-1 report.

Giorgia Caspani, Gustavo Turecki, Raymond W. Lam, Roumen V. Milev, Benicio N. Frey, Glenda M. MacQueen, Daniel J. Müller, Susan Rotzinger, Sidney H. Kennedy, Jane A. Foster, Jonathan R. Swann

Supplementary Material

Supplementary Figure 1. PCA plots of lipoprotein and urinary profiles colored by recruitment site. The lipoprotein (**a**) and urinary (**b**) metabolomes of the participants were not affected by study site, as shown by the largely overlapping distributions in the scores plots. CAM = Centre for Addiction and Mental Health; MCU = McMaster University; QNS = Queen's University; TGH = Toronto General/Western Hospital; UBC = University of British Columbia; UCA = University of Calgary.

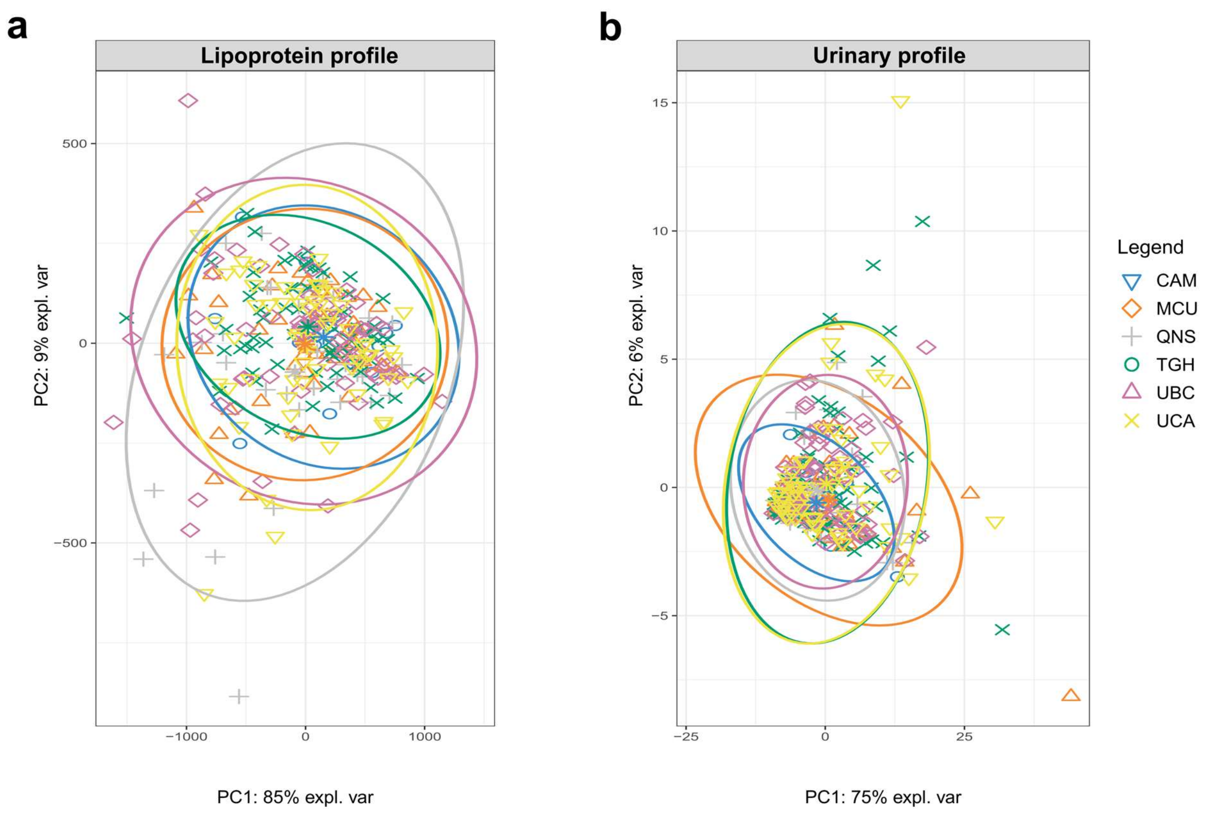

Supplementary Figure 2. Plasma metabolic clusters correlated with MADRS at baseline and covariates. Heatmap of correlations between modules of plasma lipoproteins and demographic and clinical variables in (**a**) all participants, (**b**) males, (**c**) females. For significant correlations only, the correlation coefficient and p value (in brackets) are shown. The tables show the individual plasma lipoproteins that belong to each module.

a

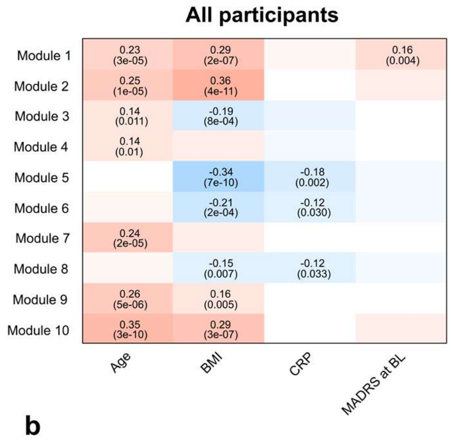

Modules	Metabolites
1	LDL6, LDL6 triglycerides, LDL6 cholesterol, LDL6 free cholesterol, LDL6 phospholipids, LDL6 apolipoprotein-B
2	Total triglycerides, VLDL, IDL, VLDL triglycerides, IDL triglycerides, LDL triglycerides, VLDL cholesterol, IDL cholesterol, VLDL free cholesterol IDL free cholesterol, VLDL phospholipids, IDL phospholipids, VLDL apolipoprotein-B, IDL apolipoprotein-B, VLDL1 triglycerides, VLDL2 triglycerides, VLDL3 triglycerides, VLDL4 triglycerides, VLDL5 triglycerides, VLDL1 cholesterol, VLDL2 cholesterol, VLDL3 cholesterol, VLDL4 cholesterol, VLDL5 cholesterol, VLDL1 free cholesterol, VLDL2 free cholesterol, VLDL3 free cholesterol, VLDL4 free cholesterol, VLDL5 free cholesterol, VLDL1 phospholipids, VLDL2 phospholipids, VLDL3 phospholipids, VLDL4 phospholipids, VLDL5 phospholipids, LDL1 triglycerides, HDL4 triglycerides
3	HDL4 cholesterol, HDL4 free cholesterol, HDL4 phospholipids, HDL4 apolipoprotein-A1, HDL4 apolipoprotein-A2
4	HDL triglycerides, HDL1 triglycerides, HDL2 triglycerides, HDL3 triglycerides
5	HDL cholesterol, Total apolipoprotein-A1, HDL free cholesterol, HDL phospholipids, HDL apolipoprotein-A1, HDL1 cholesterol, HDL2 cholesterol, HDL1 free cholesterol, HDL2 free cholesterol, HDL1 phospholipids, HDL2 phospholipids, HDL1 apolipoprotein-A1, HDL2 apolipoprotein-A1, HDL1 apolipoprotein-A2
6	Total apolipoprotein-A2, HDL apolipoprotein-A2, HDL3 cholesterol, HDL3 free cholesterol, HDL3 phospholipids, HDL3 apolipoprotein-A1, HDL2 apolipoprotein-A2, HDL3 apolipoprotein-A2
7	LDL1, LDL2 triglycerides, LDL3 triglycerides, LDL1 cholesterol, LDL1 free cholesterol, LDL1 phospholipids, LDL1 apolipoprotein-B
8	LDL2, LDL3, LDL2 cholesterol, LDL3 cholesterol, LDL2 free cholesterol, LDL3 free cholesterol, LDL2 phospholipids, LDL3 phospholipids, LDL2 apolipoprotein-B, LDL3 apolipoprotein-B
9	LDL4, LDL4 triglycerides, LDL4 cholesterol, LDL4 free cholesterol, LDL4 phospholipids, LDL4 apolipoprotein-B
10	Total cholesterol, LDL cholesterol, Total apolipoprotein-B, LDL/HDL, apo-B/apo-A1, Total particle number, LDL, LDL5, LDL free cholesterol, LDL phospholipids, LDL apolipoprotein-B, LDL5 triglycerides, LDL5 cholesterol, LDL5 free cholesterol, LDL5 phospholipids, LDL5

b

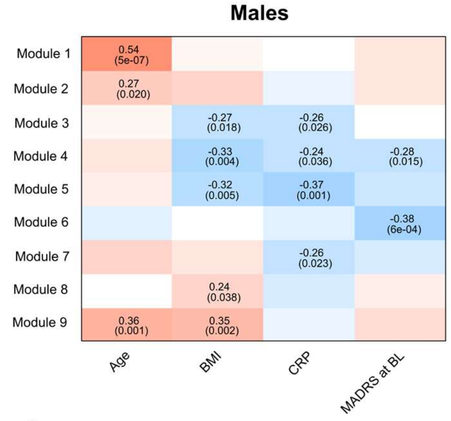

Modules	Metabolites
1	LDL6, LDL6 triglycerides, LDL6 cholesterol, LDL6 free cholesterol, LDL6 phospholipids, LDL6 apolipoprotein-B
2	Total triglycerides, VLDL, IDL, VLDL triglycerides, IDL triglycerides, LDL triglycerides, HDL triglycerides, VLDL cholesterol, IDL cholesterol, VLDL free cholesterol, IDL free cholesterol, VLDL phospholipids, IDL phospholipids, VLDL apolipoprotein-B, IDL apolipoprotein-B, VLDL1 triglycerides, VLDL2 triglycerides, VLDL3 triglycerides, VLDL4 triglycerides, VLDL5 triglycerides, VLDL1 cholesterol, VLDL2 cholesterol, VLDL3 cholesterol, VLDL4 cholesterol, VLDL5 cholesterol, VLDL1 free cholesterol, VLDL2 free cholesterol, VLDL3 free cholesterol, VLDL4 free cholesterol, VLDL5 free cholesterol, VLDL1 phospholipids, VLDL2 phospholipids, VLDL3 phospholipids, VLDL4 phospholipids, VLDL5 phospholipids, LDL1 triglycerides, LDL3 triglycerides, HDL1 triglycerides, HDL2 triglycerides, HDL3 triglycerides, HDL4 triglycerides
3	HDL4 cholesterol, HDL4 free cholesterol, HDL4 phospholipids, HDL4 apolipoprotein-A1, HDL4 apolipoprotein-A2
4	HDL cholesterol, LDL/HDL, HDL free cholesterol, HDL phospholipids, HDL1 cholesterol, HDL2 cholesterol, HDL1 free cholesterol, HDL2 free cholesterol, HDL1 phospholipids, HDL2 phospholipids, HDL1 apolipoprotein-A1, HDL2 apolipoprotein-A1, HDL1 apolipoprotein-A2, HDL2 apolipoprotein-A2
5	Total apolipoprotein-A1, Total apolipoprotein-A2, HDL apolipoprotein-A1, HDL apolipoprotein-A2, HDL3 cholesterol, HDL3 free cholesterol, HDL3 phospholipids, HDL3 apolipoprotein-A1, HDL3 apolipoprotein-A2
6	LDL1, LDL2, LDL3, LDL2 triglycerides, LDL1 cholesterol, LDL2 cholesterol, LDL3 cholesterol, LDL1 free cholesterol, LDL2 free cholesterol, LDL3 free cholesterol, LDL1 phospholipids, LDL2 phospholipids, LDL3 phospholipids, LDL1 apolipoprotein-B, LDL2 apolipoprotein-B, LDL3 apolipoprotein-B
7	Total cholesterol, LDL cholesterol, LDL, LDL free cholesterol, LDL phospholipids, LDL apolipoprotein-B
8	LDL4, LDL4 triglycerides, LDL4 cholesterol, LDL4 free cholesterol, LDL4 phospholipids, LDL4 apolipoprotein-B
9	Total apolipoprotein-B, apo-B/apo-A1, Total particle number, LDL5, LDL5 triglycerides, LDL5 cholesterol, LDL5 free cholesterol, LDL5 phospholipids, LDL5 apolipoprotein-B

c

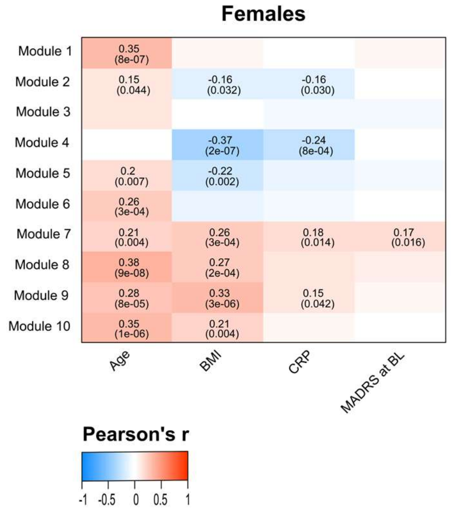

Modules	Metabolites
1	LDL1, LDL2 triglycerides, LDL3 triglycerides, LDL1 cholesterol, LDL1 free cholesterol, LDL1 phospholipids, LDL1 apolipoprotein-B
2	LDL2, LDL3, LDL2 cholesterol, LDL3 cholesterol, LDL2 free cholesterol, LDL3 free cholesterol, LDL2 phospholipids, LDL3 phospholipids, LDL2 apolipoprotein-B, LDL3 apolipoprotein-B
3	HDL triglycerides, HDL1 triglycerides, HDL2 triglycerides, HDL3 triglycerides
4	HDL cholesterol, Total apolipoprotein-A1, HDL free cholesterol, HDL phospholipids, HDL apolipoprotein-A1, HDL1 cholesterol, HDL2 cholesterol, HDL1 free cholesterol, HDL2 free cholesterol, HDL1 phospholipids, HDL2 phospholipids, HDL1 apolipoprotein-A1, HDL2 apolipoprotein-A1, HDL1 apolipoprotein-A2, HDL2 apolipoprotein-A2
5	Total apolipoprotein-A2, HDL apolipoprotein-A2, HDL3 cholesterol, HDL3 free cholesterol, HDL3 phospholipids, HDL3 apolipoprotein-A1, HDL3 apolipoprotein-A2
6	HDL4 cholesterol, HDL4 free cholesterol, HDL4 phospholipids, HDL4 apolipoprotein-A1, HDL4 apolipoprotein-A2
7	LDL6, LDL6 triglycerides, LDL6 cholesterol, LDL6 free cholesterol, LDL6 phospholipids, LDL6 apolipoprotein-B
8	Total cholesterol, LDL cholesterol, Total apolipoprotein-B, LDL/HDL, apo-B/apo-A1, Total particle number, IDL, LDL, LDL4, LDL5, LDL triglycerides, IDL cholesterol, IDL free cholesterol, LDL free cholesterol, LDL phospholipids, IDL apolipoprotein-B, LDL apolipoprotein-B, LDL1 triglycerides, LDL4 triglycerides, LDL5 triglycerides, LDL4 cholesterol, LDL5 cholesterol, LDL4 free cholesterol, LDL5 free cholesterol, LDL4 phospholipids, LDL5 phospholipids, LDL4 apolipoprotein-B, LDL5 apolipoprotein-B
9	Total triglycerides, VLDL, VLDL triglycerides, IDL triglycerides, VLDL cholesterol, VLDL free cholesterol, VLDL phospholipids, IDL phospholipids, VLDL apolipoprotein-B, VLDL1 triglycerides, VLDL2 triglycerides, VLDL3 triglycerides, VLDL4 triglycerides, VLDL1 cholesterol, VLDL2 cholesterol, VLDL3 cholesterol, VLDL4 cholesterol, VLDL1 free cholesterol, VLDL2 free cholesterol, VLDL3 free cholesterol, VLDL4 free cholesterol, VLDL5 free cholesterol, VLDL1 phospholipids, VLDL2 phospholipids, VLDL3 phospholipids, VLDL4 phospholipids, HDL4 triglycerides
10	VLDL5 triglycerides, VLDL5 cholesterol, VLDL5 phospholipids

Supplementary Figure 3. Plasma metabolic clusters correlated with MADRS reduction during phase I/II and covariates. Heatmap of correlations between modules of plasma lipoproteins and demographic and clinical variables in (**a**) all participants, (**b**) males, (**c**) females. For significant correlations only, the correlation coefficient and p value (in brackets) are shown. The tables show the individual plasma lipoproteins that belong to each module.

a

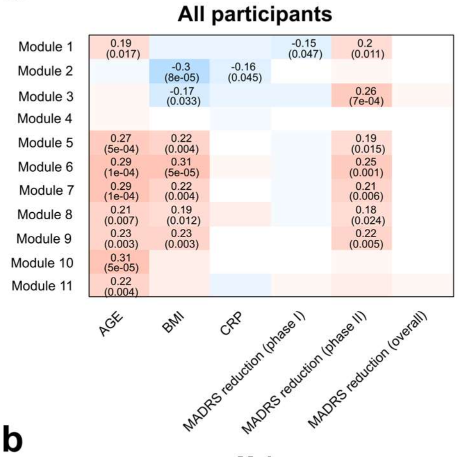

Modules	Metabolites
1	HDL4 cholesterol, HDL4 free cholesterol, HDL4 phospholipids, HDL4 apolipoprotein-A1, HDL4 apolipoprotein-A2
2	HDL cholesterol, Total apolipoprotein-A1, HDL free cholesterol, HDL phospholipids, HDL apolipoprotein-A1, HDL1 triglycerides, HDL1 cholesterol, HDL2 cholesterol, HDL1 free cholesterol, HDL2 free cholesterol, HDL1 phospholipids, HDL2 phospholipids, HDL1 apolipoprotein-A1, HDL2 apolipoprotein-A1, HDL1 apolipoprotein-A2, HDL2 apolipoprotein-A2
3	Total apolipoprotein-A2, HDL apolipoprotein-A2, HDL3 cholesterol, HDL3 free cholesterol, HDL3 phospholipids, HDL3 apolipoprotein-A1, HDL3 apolipoprotein-A2
4	LDL1, LDL2, LDL3, LDL2 triglycerides, LDL3 triglycerides, LDL1 cholesterol LDL2 cholesterol, LDL3 cholesterol, LDL1 free cholesterol, LDL2 free cholesterol, LDL3 free cholesterol, LDL1 phospholipids, LDL2 phospholipids, LDL3 phospholipids, LDL1 apolipoprotein-B, LDL2 apolipoprotein-B, LDL3 apolipoprotein-B
5	LDL4, LDL4 triglycerides, LDL4 cholesterol, LDL4 free cholesterol, LDL4 phospholipids, LDL4 apolipoprotein-B
6	LDL/HDL, apo-B/apo-A1, LDL5, LDL5 triglycerides, LDL5 cholesterol, LDL5 free cholesterol, LDL5 phospholipids, LDL5 apolipoprotein-B
7	Total cholesterol, LDL cholesterol, Total apolipoprotein-B, Total particle number, LDL, LDL triglycerides, LDL free cholesterol, LDL phospholipids, LDL apolipoprotein-B, LDL1 triglycerides
8	LDL6, LDL6 triglycerides, LDL6 cholesterol, LDL6 free cholesterol, LDL6 phospholipids, LDL6 apolipoprotein-B
9	Total triglycerides, VLDL, IDL, VLDL triglycerides, IDL triglycerides, VLDL cholesterol, IDL cholesterol, VLDL free cholesterol, IDL free cholesterol, VLDL phospholipids, IDL phospholipids, VLDL apolipoprotein-B, IDL apolipoprotein-B, VLDL1 triglycerides, VLDL2 triglycerides, VLDL3 triglycerides, VLDL4 triglycerides, VLDL1 cholesterol, VLDL2 cholesterol, VLDL3 cholesterol, VLDL4 cholesterol, VLDL1 free cholesterol, VLDL2 free cholesterol, VLDL3 free cholesterol, VLDL4 free cholesterol, VLDL5 free cholesterol, VLDL1 phospholipids, VLDL2 phospholipids, VLDL3 phospholipids, VLDL4 phospholipids, HDL4 triglycerides
10	VLDL5 triglycerides, VLDL5 cholesterol, VLDL5 phospholipids
11	HDL triglycerides, HDL2 triglycerides, HDL3 triglycerides

b

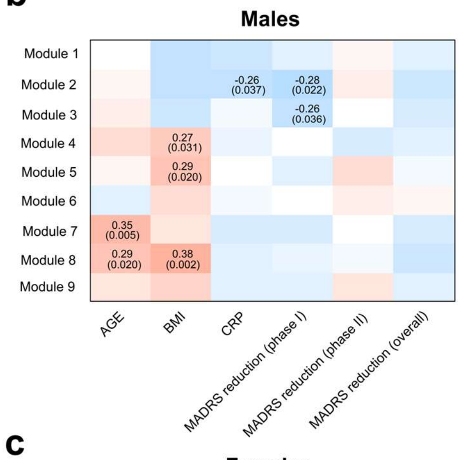

Modules	Metabolites
1	HDL4 cholesterol, HDL4 free cholesterol, HDL4 phospholipids, HDL4 apolipoprotein-A1, HDL4 apolipoprotein-A2,
2	HDL cholesterol, Total apolipoprotein-A1, Total apolipoprotein-A2, HDL phospholipids, HDL apolipoprotein-A1, HDL apolipoprotein-A2, HDL2 cholesterol, HDL3 cholesterol, HDL2 free cholesterol, HDL3 free cholesterol, HDL2 phospholipids, HDL3 phospholipids, HDL2 apolipoprotein-A1, HDL3 apolipoprotein-A1, HDL1 apolipoprotein-A2, HDL2 apolipoprotein-A2, HDL3 apolipoprotein-A2
3	HDL free cholesterol, HDL1 cholesterol, HDL1 free cholesterol, HDL1 phospholipids, HDL1 apolipoprotein-A1
4	LDL3, LDL4, LDL4 triglycerides, LDL3 cholesterol, LDL4 cholesterol, LDL3 free cholesterol, LDL4 free cholesterol, LDL3 phospholipids, LDL4 phospholipids, LDL3 apolipoprotein-B, LDL4 apolipoprotein-B
5	LDL1, LDL2 triglycerides, LDL1 cholesterol, LDL1 free cholesterol, LDL1 phospholipids, LDL1 apolipoprotein-B
6	LDL2, LDL2 cholesterol, LDL2 free cholesterol, LDL2 phospholipids, LDL2 apolipoprotein-B
7	HDL triglycerides, HDL1 triglycerides, HDL2 triglycerides
8	Total cholesterol, LDL cholesterol, Total apolipoprotein-B, LDL/HDL, apo-B/apo-A1 Total particle number, LDL, LDL5, LDL free cholesterol, LDL phospholipids, LDL apolipoprotein-B, LDL3 triglycerides, LDL5 triglycerides, LDL5 cholesterol, LDL5 free cholesterol, LDL5 phospholipids, LDL5 apolipoprotein-B
9	Total triglycerides, VLDL, IDL, LDL6, VLDL triglycerides, IDL triglycerides, LDL triglycerides, VLDL cholesterol, IDL cholesterol, VLDL free cholesterol, IDL free cholesterol, VLDL phospholipids, IDL phospholipids, VLDL apolipoprotein-B, IDL apolipoprotein-B, VLDL1 triglycerides, VLDL2 triglycerides, VLDL3 triglycerides, VLDL4 triglycerides, VLDL5 triglycerides, VLDL1 cholesterol, VLDL2 cholesterol, VLDL3 cholesterol, VLDL4 cholesterol, VLDL5 cholesterol, VLDL1 free cholesterol, VLDL2 free cholesterol, VLDL3 free cholesterol VLDL4 free cholesterol, VLDL5 free cholesterol, VLDL1 phospholipids, VLDL2 phospholipids, VLDL3 phospholipids, VLDL4 phospholipids, VLDL5 phospholipids, LDL1 triglycerides, LDL6 triglycerides, LDL6 cholesterol, LDL6 free cholesterol, LDL6 phospholipids, LDL6 apolipoprotein-B, HDL3 triglycerides, HDL4 triglycerides

c

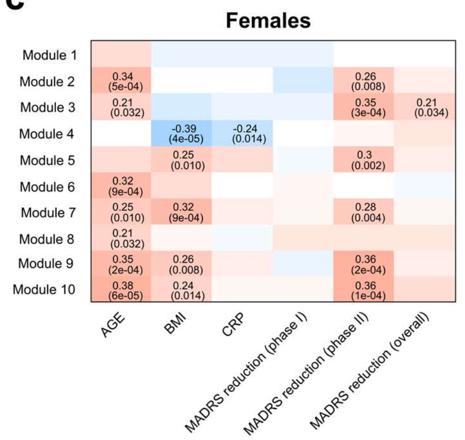

Modules	Metabolites
1	LDL1, LDL2, LDL3, LDL free cholesterol, LDL2 triglycerides, LDL3 triglycerides, LDL1 cholesterol, LDL2 cholesterol, LDL3 cholesterol, LDL1 free cholesterol, LDL2 free cholesterol, LDL3 free cholesterol, LDL1 phospholipids, LDL2 phospholipids, LDL3 phospholipids, LDL1 apolipoprotein-B, LDL2 apolipoprotein-B, LDL3 apolipoprotein-B
2	HDL4 cholesterol, HDL4 free cholesterol, HDL4 phospholipids, HDL4 apolipoprotein-A1, HDL4 apolipoprotein-A2
3	Total apolipoprotein-A2, HDL apolipoprotein-A2, HDL3 cholesterol, HDL3 free cholesterol, HDL3 phospholipids, HDL3 apolipoprotein-A1, HDL3 apolipoprotein-A2
4	HDL cholesterol, Total apolipoprotein-A1, HDL free cholesterol, HDL phospholipids, HDL apolipoprotein-A1, HDL1 triglycerides, HDL1 cholesterol, HDL2 cholesterol, HDL1 free cholesterol, HDL2 free cholesterol, HDL1 phospholipids, HDL2 phospholipids, HDL1 apolipoprotein-A1, HDL2 apolipoprotein-A1, HDL1 apolipoprotein-A2, HDL2 apolipoprotein-A2
5	LDL6, LDL6 triglycerides, LDL6 cholesterol, LDL6 free cholesterol, LDL6 phospholipids, LDL6 apolipoprotein-B
6	VLDL5 triglycerides, VLDL5 cholesterol, VLDL5 free cholesterol, VLDL5 phospholipids
7	Total triglycerides, VLDL, VLDL triglycerides, IDL triglycerides, VLDL cholesterol, VLDL free cholesterol, VLDL phospholipids, IDL phospholipids, VLDL apolipoprotein-B, VLDL1 triglycerides, VLDL2 triglycerides, VLDL3 triglycerides, VLDL4 triglycerides, VLDL1 cholesterol, VLDL2 cholesterol, VLDL3 cholesterol, VLDL4 cholesterol, VLDL1 free cholesterol, VLDL2 free cholesterol, VLDL3 free cholesterol, VLDL4 free cholesterol, VLDL1 phospholipids, VLDL2 phospholipids, VLDL3 phospholipids, VLDL4 phospholipids, HDL4 triglycerides
8	HDL triglycerides, HDL2 triglycerides, HDL3 triglycerides
9	Total cholesterol, LDL cholesterol, Total apolipoprotein-B, LDL/HDL, apo-B/apo-A1 Total particle number, IDL, LDL, LDL4, LDL5, IDL cholesterol, IDL free cholesterol, LDL phospholipids, IDL apolipoprotein-B, LDL apolipoprotein-B, LDL5 triglycerides, LDL4 cholesterol, LDL5 cholesterol, LDL4 free cholesterol, LDL5 free cholesterol, LDL4 phospholipids, LDL5 phospholipids, LDL4 apolipoprotein-B, LDL5 apolipoprotein-B
10	LDL triglycerides, LDL1 triglycerides, LDL4 triglycerides

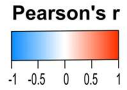

Supplementary Figure 4. Scores plots from the PCA models constructed on the urinary metabolic and plasma lipoprotein profiles of study samples and quality control samples. Scores plots showing the (**a**) lipoprotein profiles and (**b**) urinary metabolic profiles of all study samples (orange) and the pooled quality control (QC) samples (blue).

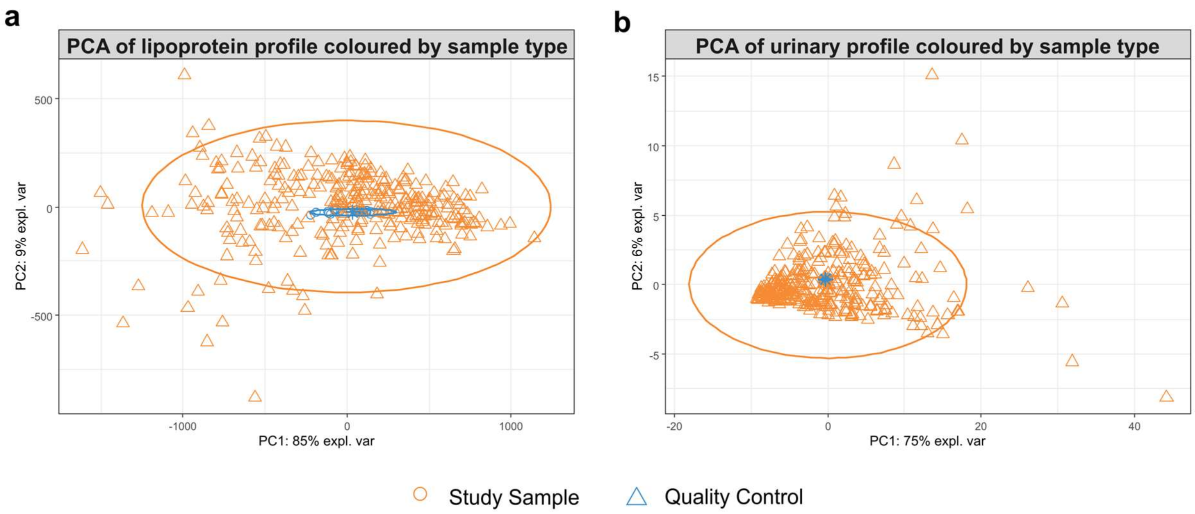

Supplementary Table 1. MADRS scores and circulating CRP concentrations in male and female MDD participants across timepoints. Values are mean ± standard deviation for escitalopram and aripiprazole responders and non-responders at baseline, week 8 and week 16. Data is shown for males and females separately. * indicates one missing observation; ** indicates two missing observations.

			Escitalopram		Aripiprazole	
			Responders	Non-Responders	Responders	Non-Responders
**Males**	**MADRS**					
		Baseline	29.8 ± 5.5	29.6 ± 5.2	30.8 ± 5.1	28.4 ± 5.3
		Week 8	8.1 ± 5.2	24.5 ± 7.3	23.3 ± 6.0	26.4 ± 8.6
		Week 16	5.6 ± 4.7	15.4 ± 8.9	9.9 ± 5.0	21.6 ± 7.6
	**CRP**					
		Baseline	4.3 ± 7.9	1.5 ± 2.2 *	1.2 ± 1.3	1.8 ± 3.0
		Week 8	2.6 ± 3.2 *	4.2 ± 16.2	1.5 ± 2.6	7.3 ± 23.7
		Week 16	5.5 ± 11.0	2.0 ± 2.4	1.8 ± 2.5	2.2 ± 2.4
**Females**	**MADRS**					
		Baseline	29.2 ± 5.5	30.5 ± 5.7	31.2 ± 6.0	29.9 ± 5.6
		Week 8	8.1 ± 5.0	23.1 ± 7.3	21.6 ± 5.5	25.1 ± 9.2
		Week 16	6.7 ± 7.3	13.6 ± 8.5 *	8.4 ± 4.8 *	22.1 ± 6.0
	**CRP**					
		Baseline	3.7 ± 6.1 *	4.5 ± 10.3 *	4.4 ± 11.3	5.1 ± 9.6 *
		Week 8	3.2 ± 4.3 *	2.6 ± 3.6	2.0 ± 2.0	3.8 ± 5.3
		Week 16	3.5 ± 4.8	3.8 ± 6.6 **	3.9 ± 7.7 *	3.7 ± 4.4 *
