# Supplementary material for: Metabolomic signatures associated with depression and predictors of antidepressant response in humans: A CAN-BIND-1 report
Source: Commun Biol. 2021 Jul 22;4:903. doi: 10.1038/s42003-021-02421-6 (PMC8298446; doi:10.1038/s42003-021-02421-6)
Supplement: Supplementary file 9 — Reporting Summary [file 42003_2021_2421_MOESM9_ESM.pdf]

nature research

Corresponding author(s): Jonathan Swann

Last updated by author(s): May 7, 2021

Reporting Summary

Nature Research wishes to improve the reproducibility of the work that we publish. This form provides structure for consistency and transparency in reporting. For further information on Nature Research policies, see our Editorial Policies and the Editorial Policy Checklist.

Statistics

For all statistical analyses, confirm that the following items are present in the figure legend, table legend, main text, or Methods section.

n/a	Confirmed	
☐	☒	The exact sample size (*n*) for each experimental group/condition, given as a discrete number and unit of measurement
☐	☒	A statement on whether measurements were taken from distinct samples or whether the same sample was measured repeatedly
☐	☒	The statistical test(s) used AND whether they are one- or two-sided<br>*Only common tests should be described solely by name; describe more complex techniques in the Methods section.*
☐	☒	A description of all covariates tested
☐	☒	A description of any assumptions or corrections, such as tests of normality and adjustment for multiple comparisons
☐	☒	A full description of the statistical parameters including central tendency (e.g. means) or other basic estimates (e.g. regression coefficient) AND variation (e.g. standard deviation) or associated estimates of uncertainty (e.g. confidence intervals)
☐	☒	For null hypothesis testing, the test statistic (e.g. *F*, *t*, *r*) with confidence intervals, effect sizes, degrees of freedom and *P* value noted<br>*Give P values as exact values whenever suitable.*
☒	☐	For Bayesian analysis, information on the choice of priors and Markov chain Monte Carlo settings
☐	☒	For hierarchical and complex designs, identification of the appropriate level for tests and full reporting of outcomes
☐	☒	Estimates of effect sizes (e.g. Cohen's *d*, Pearson's *r*), indicating how they were calculated

Our web collection on statistics for biologists contains articles on many of the points above.

Software and code

Policy information about availability of computer code

Data collection	nPYc-Toolbox version 1.0.0 for the import, quality-control, and preprocessing of metabolic profiling datasets
Data analysis	No custom software/code was used. Here is a list of packages used for data analysis:<br>Weighted gene correlation network analysis: R package 'WGCNA' version 1.69<br>Mann-Whitney U test: Matlab 'ranksum()'<br>Benjamini-Hochberg correction: Matlab 'fdr_bh' version 2.3.0.0<br>Logistic regression: R package 'glm.predict' version 3.1.0<br>AUC: R package 'pROC' version 1.16.2<br>Partial correlation: R package 'ppcor' version 1.1<br>Spearman correlation: R package 'stats' version 4.0.1

For manuscripts utilizing custom algorithms or software that are central to the research but not yet described in published literature, software must be made available to editors and reviewers. We strongly encourage code deposition in a community repository (e.g. GitHub). See the Nature Research guidelines for submitting code & software for further information.

Data

Policy information about availability of data

All manuscripts must include a data availability statement. This statement should provide the following information, where applicable:

- Accession codes, unique identifiers, or web links for publicly available datasets
- A list of figures that have associated raw data
- A description of any restrictions on data availability

The data produced and used in this study are supported by The Canadian Biomarker Integration Network in Depression (CAN-BIND, https://canbind.ca/), which is an

Integrative Discovery Program funded by the Ontario Brain Institute (OBI, https://braininstitute.ca/). In accordance with the Research Activity Agreements between CAN-BIND and OBI, data produced in this study must be submitted to Brain-CODE (https://www.braincode.ca/), an informatic platform created by the OBI to facilitate open-access of data generated from research funded by the OBI.

Researchers requesting Data will provide OBI with written documentation of the proposed use of the Data in the form of an Research Ethics Board (REB) approval package which includes the full REB submission package and REB approval letter from their local Institutional REB. Should the REB determine that ethics review is not required, an exemption letter from the REB will be required. If the External Researcher is from an Institution that does not have a local REB, OBI will work with the External Researcher to identify a mutually acceptable REB for review. All documents not in English or French will require a certified translation copy.

For detailed data access policy and procedure, please refer https://www.braininstitute.ca/research-data-sharing/brain-code or contact help@braincode.ca

Field-specific reporting

Please select the one below that is the best fit for your research. If you are not sure, read the appropriate sections before making your selection.

☒ Life sciences ☐ Behavioural & social sciences ☐ Ecological, evolutionary & environmental sciences

For a reference copy of the document with all sections, see nature.com/documents/nr-reporting-summary-flat.pdf

Life sciences study design

All studies must disclose on these points even when the disclosure is negative.

Sample size	The initial recruitment target was set to 200 for participants with Major Depressive Disorder and 90 for healthy controls across 6 recruitment sites. The selected sample size was based on the analytic plan to identify standalone features that differ significantly between treatment responders and non-responders, either at baseline or over the course of the study. Parametric two-sample t-tests will be used for features with normally distributed data. Otherwise, the non-parametric Mann–Whitney U test will be used. A significance threshold of α = 0.05 and multiple testing corrections will be required. Given an input list of up to 25,000 features, of which at least 250 are differentially expressed between responders and non-responders, assuming an overall false discovery rate of 5 %, and applying two-sample t-testing procedures, a minimum of 49 subjects per category are required for power of at least 90 % to correctly identify a given feature which differs significantly between the categories. If nonparametric testing procedures are used, a minimum of 52 subjects per category are required to achieve the same power. Target recruitment for patients with MDD is 200. An estimated response rate of 60 % would yield 120 responders and 80 non-responders. The planned sample size is sufficiently powered for both parametric and non-parametric univariate testing. Multivariate analysis and the development of prognostic signatures of escitalopram response will follow univariate analysis.
Data exclusions	1 participant was excluded due to an incidental finding discovered by Magnetic resonance imaging. After consultation with the radiologist, the CAN-BIND clinical team decided that the participant should have been screened out based on a prior diagnosis, and the data (clinical, imaging, EEG, molecular) should therefore not be included in any analyses.
Replication	All tsamples were analysed following strict protocols, published in Dona et a. (2014)*, that cover all the steps in the analysis, from sample collection, preparation, and NMR data acquisition. The validity and reproducibility of our NMR-based findings were verified by running quality control (QC) samples, referred to 'study reference' (SR) in the manuscript, along with the study samples. QC samples provide an idea of the total variation across the preparation and analysis stages (i.e. intra-study variation). QC samples were prepared by pooling equal parts of each study sample and were acquired every 40 study samples throughout the analysis. The visualisation of a PCA model built on all samples showed that the data points representing the QC clustered together at the origin, confirming that the variation observed in the study samples is reflective of biological and not analytical (i.e. sample preparation) differences. This was confirmed for both urine and plasma samples.<br>The analytical platforms used in this study are highly reliable and reproducible. In particular, exceptional reproducibility was demonstrated for the lipoprotein subclass analysis used in this study (Jiménez et al., 2018)**, supporting its applicability for clinical purposes.<br><br>*Dona, A. C. et al. Precision High-Throughput Proton NMR Spectroscopy of Human Urine, Serum, and Plasma for Large-Scale Metabolic Phenotyping. Anal. Chem. 86, 9887–9894 (2014).<br>**Jiménez, B. et al. Quantitative Lipoprotein Subclass and Low Molecular Weight Metabolite Analysis in Human Serum and Plasma by 1H NMR Spectroscopy in a Multilaboratory Trial. Anal. Chem. 90, 20, 11962–11971 (2018).
Randomization	Randomisation was also performed while running the plasma and urine samples on NMR (i.e. samples were run in a random order).
Blinding	The experimenters were blind to the identity of the samples during sample preparation, during the NMR run and pre-processing. The analysis of the data required the authors to know the participant's diagnosis, what treatment arm the MDD participants followed, and whether treatment was successful. However, the nature of the analysis performed did not allow for external biases (i.e. the same statistical workflow was used on all data from all participants).

Reporting for specific materials, systems and methods

We require information from authors about some types of materials, experimental systems and methods used in many studies. Here, indicate whether each material, system or method listed is relevant to your study. If you are not sure if a list item applies to your research, read the appropriate section before selecting a response.

Materials & experimental systems

n/a	Involved in the study
☒	☐ Antibodies
☒	☐ Eukaryotic cell lines
☒	☐ Palaeontology and archaeology
☒	☐ Animals and other organisms
☐	☒ Human research participants
☐	☒ Clinical data
☒	☐ Dual use research of concern

Methods

n/a	Involved in the study
☒	☐ ChIP-seq
☒	☐ Flow cytometry
☒	☐ MRI-based neuroimaging

Human research participants

Policy information about studies involving human research participants

Population characteristics

A total of 323 (204 females and 119 males) MDD and HC participants were recruited from 6 outpatient centers across Canada between August 2013 and December 2016112 HC participants (MADRS=0.8±1.7) and 211 MDD participants (MADRS=29.9 ±5.6) were identified. The groups were matched for age (N=323, Mann-Whitney 93 U=17229.5, p=0.252) and sex (N=323, χ (1)=0.0041; p=0.9491).

Inclusion Criteria for Depressed patients:
Outpatients who are 18-60 years of age
Meet DSM-IV-TR criteria for Major Depressive Episode in Major Depressive Disorder by the MINI
Episode duration ≥ 3 months
Free of psychotropic medications for at least 5 half-lives (i.e. 1 week for most antidepressants, 5 weeks for fluoxetine) before baseline Visit 1
MADRS ≥ 24
Fluency in English, sufficient to complete the interviews and self-report questionnaires

Exclusion Criteria for Depressed patients:
Any Axis I diagnosis other than MDD that is considered the primary diagnosis
Bipolar I or Bipolar II diagnosis
Presence of a significant Axis II diagnosis (borderline, antisocial)
High suicidal risk, defined by clinician judgment
Substance dependence/abuse in the past 6 months
Presence of significant neurological disorders, head trauma or other unstable medical conditions
Pregnant or breastfeeding
Failure of 3 or more adequate pharmacologic interventions (as determined by the Antidepressant Treatment History Form)
Started psychological treatment within the past 3 months with the intent of continuing treatment
Patients who have previously failed escitalopram or showed intolerance to escitalopram and patients at risk for hypomanic switch (i.e. with a history of antidepressant hypomania)

Inclusion criteria for Healthy Controls:
18 to 60 years of age
No history of Axis I or Axis II disorders, as determined by the MINI.
Fluency in English, sufficient to complete the interviews and self-report questionnaires.

The clinical characteristics of the patient cohort is described in details in the following paper:
Lam RW, Milev R, Rotzinger S, Andreazza AC, Blier P, Brenner C, Daskalakis ZJ, Dharsee M, Downar J, Evans KR, Farzan F, Foster JA, Frey BN, Geraci J, Giacobbe P, Feilotter HE, Hall GB, Harkness KL, Hassel S, Ismail Z, Leri F, Liotti M, MacQueen GM, McAndrews MP, Minuzzi L, Müller DJ, Parikh SV, Placenza FM, Quilty LC, Ravindran AV, Salomons TV, Soares CN, Strother SC, Turecki G, Vaccarino AL, Vila-Rodriguez F, Kennedy SH; CAN-BIND Investigator Team. Discovering biomarkers for antidepressant response: protocol from the Canadian biomarker integration network in depression (CAN-BIND) and clinical characteristics of the first patient cohort. BMC Psychiatry. 2016 Apr 16;16:105. doi: 10.1186/s12888-016-0785-x. PMID: 27084692; PMCID: PMC4833905.

Recruitment

Participants are recruited at 6 clinical centres: Vancouver (Djavad Mowafaghian Centre for Brain Health), Calgary (Hotchkiss Brain Institute), Toronto (2 sites: University Health Network and Centre for Addiction and Mental Health), Hamilton (St. Joseph's Healthcare Hamilton), and Kingston (Providence Care, Mental Health Services). Recruitment draws upon outpatient-referral networks, community-based advertising, and dedicated knowledge translation (KT) activities.

Ethics oversight

The study was conducted in compliance with the principles of Good Clinical Practice and relevant institutional research ethics boards.

Note that full information on the approval of the study protocol must also be provided in the manuscript.

Clinical data

Policy information about clinical studies

All manuscripts should comply with the ICMJE guidelines for publication of clinical research and a completed CONSORT checklist must be included with all submissions.

Clinical trial registration	ClinicalTrials.gov Identifier: NCT01655706
Study protocol	Lam RW, Milev R, Rotzinger S, Andreazza AC, Blier P, Brenner C, Daskalakis ZJ, Dharsee M, Downar J, Evans KR, Farzan F, Foster JA, Frey BN, Geraci J, Giacobbe P, Feilotter HE, Hall GB, Harkness KL, Hassel S, Ismail Z, Leri F, Liotti M, MacQueen GM, McAndrews MP, Minuzzi L, Müller DJ, Parikh SV, Placenza FM, Quilty LC, Ravindran AV, Salomons TV, Soares CN, Strother SC, Turecki G, Vaccarino AL, Vila-Rodriguez F, Kennedy SH; CAN-BIND Investigator Team. Discovering biomarkers for antidepressant response: protocol from the Canadian biomarker integration network in depression (CAN-BIND) and clinical characteristics of the first patient cohort. BMC Psychiatry. 2016 Apr 16;16:105. doi: 10.1186/s12888-016-0785-x. PMID: 27084692; PMCID: PMC4833905.
Data collection	Recruitment took place between August 2013 and December 2016 at the following locations:<br>University of Calgary, Canada, British Columbia<br>University of British Columbia, Canada, Ontario<br>McMaster University, Canada, Ontario<br>Queen's University, Canada, Ontario<br>University Health Network, Canada, Ontario<br>Centre for Addiction and Mental Health, Canada, Ontario<br><br>Strict Standard Operating Procedures (SOPs) were in place to ensure maximal consistency and reproducibility across recruitment sites, and sample collection protocols have been published in:<br><br>Lam, R. W. et al. Discovering biomarkers for antidepressant response: Protocol from the Canadian biomarker integration network in depression (CAN-BIND) and clinical characteristics of the first patient cohort. BMC Psychiatry 16, 1–13 (2016).
Outcomes	Primary Outcome Measures:<br>Change in MADRS (Montgomery-Asberg Depression Rating Scale) scores from baseline [Time Frame: Week 8, Week 16]<br>Clinical response (≥ 50% reduction in MADRS scores from baseline)
