# Supplementary material for: Metabolomic signatures associated with depression and predictors of antidepressant response in humans: A CAN-BIND-1 report
Source: Commun Biol. 2021 Jul 22;4:903. doi: 10.1038/s42003-021-02421-6 (PMC8298446; doi:10.1038/s42003-021-02421-6)
Supplement: Supplementary file 11 — Description of Additional Supplementary Files [file 42003_2021_2421_MOESM11_ESM.pdf]

Description of Additional Supplementary Files

File name: Supplementary Data 1

Description: B.I.-LISA lipoprotein panel. The table summarizes information related to the lipoproteins measured in the present study.

File name: Supplementary Data 2

Description: B. B.I.QUANT-UR panel. The table summarizes information related to the urinary compounds measured in the present study. LOD (limit of detection)

File name: Supplementary Data 3

Description: *Fold-change of metabolites pre- and post-treatment for male responders and non-responders to escitalopram.* The metabolites presented were selected on the basis of the WGCNA analysis. Module membership represents the correlation of an individual metabolite with its module eigengene. Fold change was calculated as the log2 of the ratio between metabolite concentration at week 8 over baseline. Thus, positive values indicate an increase and negative values indicate a decrease. The p value refers to the Mann-Whitney U test, with asterisks indicating the discriminatory metabolites between escitalopram responders and non-responders.

File name: Supplementary Data 4

Description: Fold-change of metabolites pre- and post-treatment for male responders and non-responders to escitalopram. The metabolites presented were selected on the basis of the WGCNA analysis. Module membership represents the correlation of an individual metabolite with its module eigengene. Fold change was calculated as the log2 of the ratio between metabolite concentration at week 8 over baseline. Thus, positive values indicate an increase and negative values indicate a decrease. The p value refers to the Mann-Whitney U test, with asterisks indicating the discriminatory metabolites between escitalopram responders and non-responders.
